# Laryngeal Trauma Complicating a Suicide Attempt by Hanging

**DOI:** 10.5334/jbr-btr.969

**Published:** 2016-01-28

**Authors:** Pierre Tritschler, Gilles Delahaut, Xavier Pavard

**Affiliations:** 1 Cliniques Universitaires Saint Luc Bruxelles, BE

A 40-year-old man was admitted to the emergency room after a suicide attempt. He was found hanged in his garden shelter by a neighbour. The patient complained only of a slight dysphagia and anterior cervical pain. The ­clinical examination revealed no cutaneous lesion or neurological deficit. Head and neck CT scan with and without injection of contrast agent was performed. CT has shown ­displaced fractures of the two horns of the thyroïd cartilage (Figs. A, B; white arrow). Fractures were associated with a small tumefaction of the left hypopharyngeal soft tissue (Fig. C; white star) and left side of the larynx. The airways were patent. The ENT specialist achieved a ­laryngoscopy and confirmed the small buldging of the larynx, with no significant effect on the airways. Larynx mobility was conserved. A control fibroscopy was done 12 hours later with no modification of the lesions.

**Figure A:**
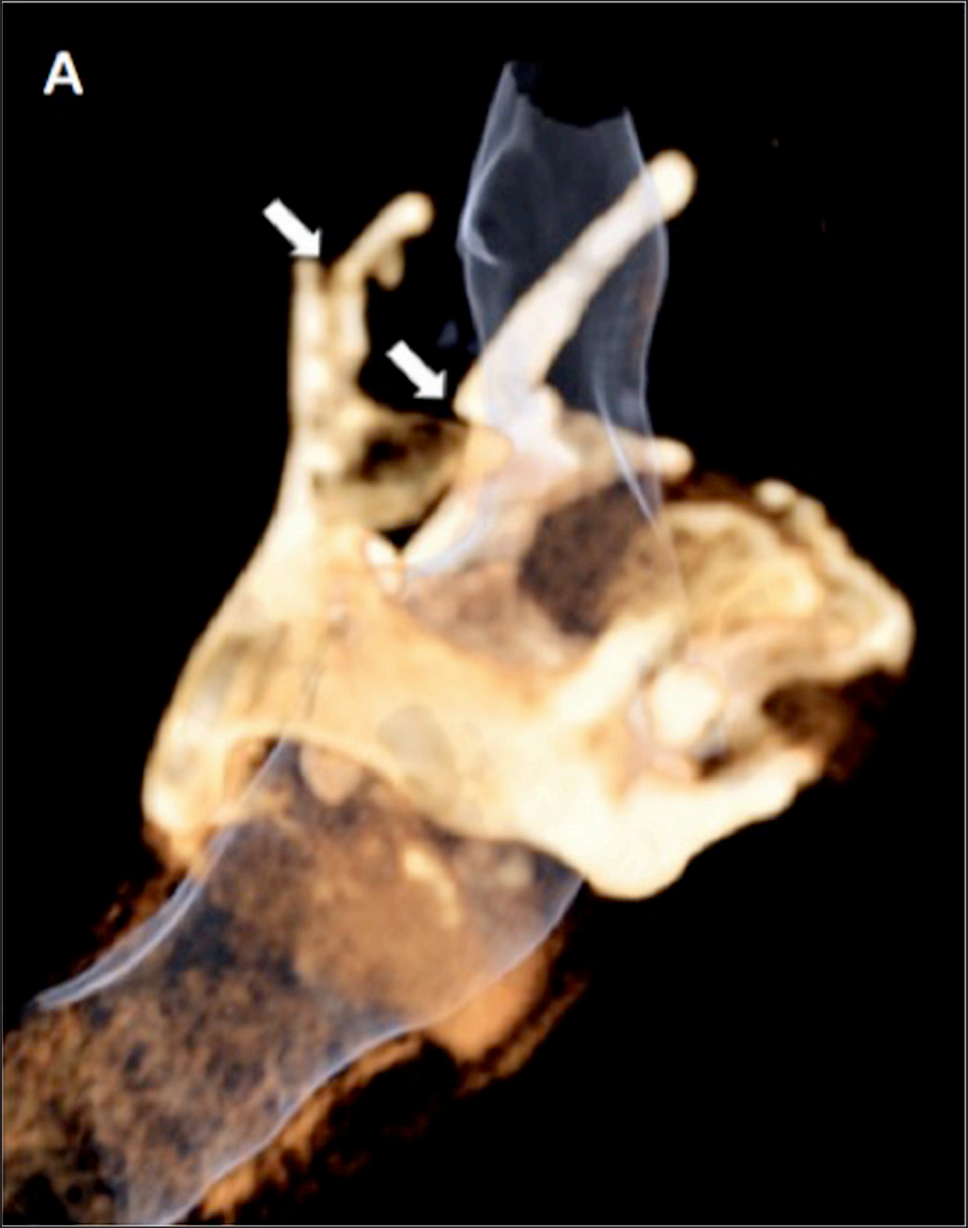


**Figure B:**
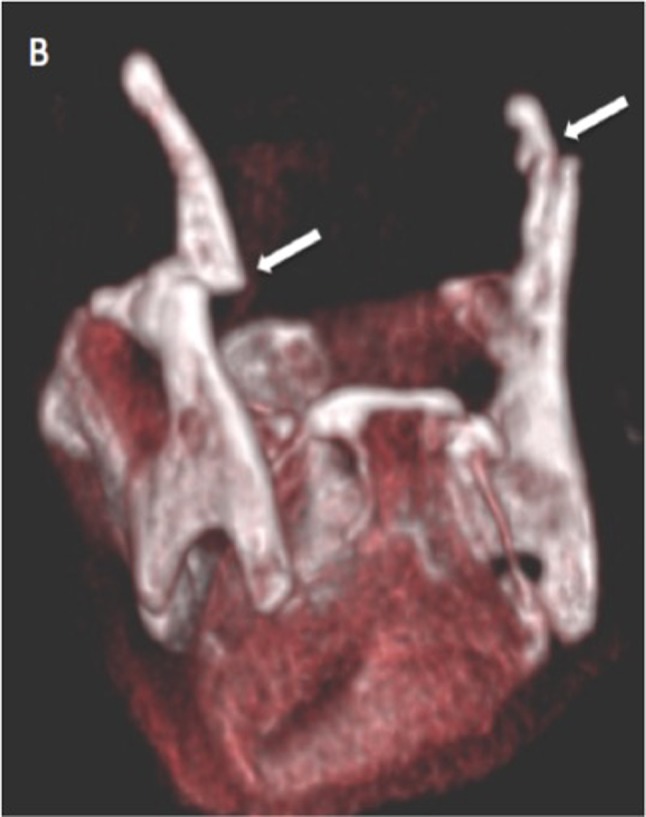


**Figure C:**
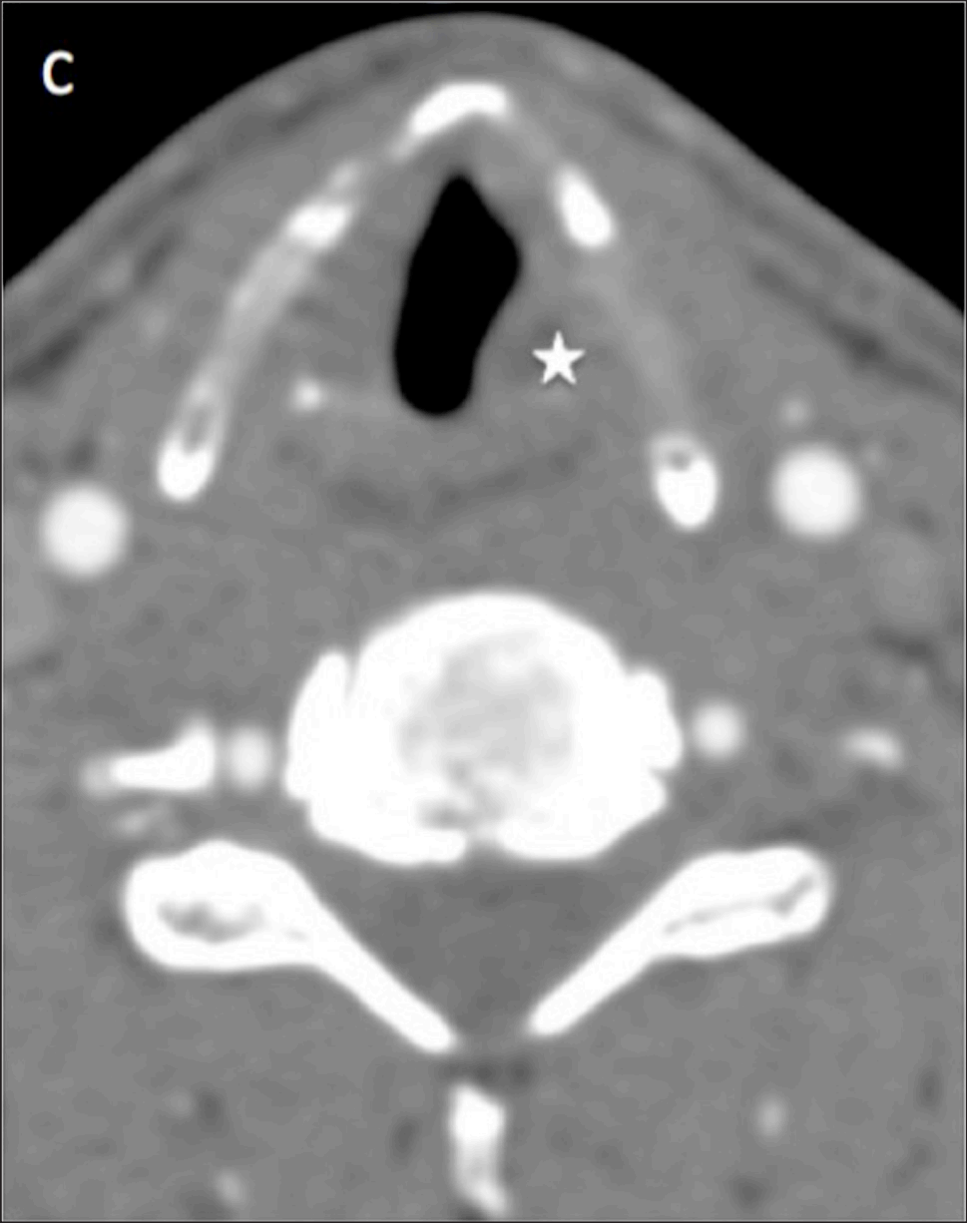


Vascular structures were patent, without thrombosis or dissection. No bone lesion of cervical spine was found. Management consisted of conservative treatment and psychatric support.

## Comment

Fractures of the larynx are uncommon trauma that are associated with other life threatening injuries in about 45% of cases. The thyroid cartilage is the most often affected structure and horizontal tears are classically detected in strangulation cases. They are classically bilateral in the thyroid cartilage and affect the superior edge of the lamina and the superior horns [1]. These kind of fractures are often combined with hyoid bone tears and hypopharyngeal hematoma. 3D volume rendering is a good tool in association with axial CT images for the detection of these fractures. The diagnosis of fracture can be difficult because of the progressive ossification of the thyroïd cartilage and differs from one person to another. The diagnosis is probably undervalued which can cause complications. Patient with nondisplaced laryngeal fractures can have long-term voice change if they are not treated. Butler et al reported that the voice results of a patient who were treated early after injury had a recovery rate that was more than two times than patients who have delayed treatment. Displaced thyroid and cricoid cartilage tears should be surgicaly reduced et fixed. To conclude, MDCT (2D and 3D VR) is crucial tool to detect quickly laryngeal injuries and other associated injuries.

## Competing Interests

The authors declare that they have no competing interests.
